# Analysis of intra-host genetic diversity of *Prunus necrotic ringspot virus* (PNRSV) using amplicon next generation sequencing

**DOI:** 10.1371/journal.pone.0179284

**Published:** 2017-06-20

**Authors:** Wycliff M. Kinoti, Fiona E. Constable, Narelle Nancarrow, Kim M. Plummer, Brendan Rodoni

**Affiliations:** 1 Agriculture Victoria, AgriBio, La Trobe University, Melbourne, VIC, Australia; 2 School of Applied Systems Biology, AgriBio, La Trobe University, Melbourne, VIC, Australia; 3 Department of Animal, Plant and Soil Sciences, AgriBio, La Trobe University, Melbourne, VIC, Australia; Oklahoma State University, UNITED STATES

## Abstract

PCR amplicon next generation sequencing (NGS) analysis offers a broadly applicable and targeted approach to detect populations of both high- or low-frequency virus variants in one or more plant samples. In this study, amplicon NGS was used to explore the diversity of the tripartite genome virus, *Prunus necrotic ringspot virus* (PNRSV) from 53 PNRSV-infected trees using amplicons from conserved gene regions of each of PNRSV RNA1, RNA2 and RNA3. Sequencing of the amplicons from 53 PNRSV-infected trees revealed differing levels of polymorphism across the three different components of the PNRSV genome with a total number of 5040, 2083 and 5486 sequence variants observed for RNA1, RNA2 and RNA3 respectively. The RNA2 had the lowest diversity of sequences compared to RNA1 and RNA3, reflecting the lack of flexibility tolerated by the replicase gene that is encoded by this RNA component. Distinct PNRSV phylo-groups, consisting of closely related clusters of sequence variants, were observed in each of PNRSV RNA1, RNA2 and RNA3. Most plant samples had a single phylo-group for each RNA component. Haplotype network analysis showed that smaller clusters of PNRSV sequence variants were genetically connected to the largest sequence variant cluster within a phylo-group of each RNA component. Some plant samples had sequence variants occurring in multiple PNRSV phylo-groups in at least one of each RNA and these phylo-groups formed distinct clades that represent PNRSV genetic strains. Variants within the same phylo-group of each *Prunus* plant sample had ≥97% similarity and phylo-groups within a *Prunus* plant sample and between samples had less ≤97% similarity. Based on the analysis of diversity, a definition of a PNRSV genetic strain was proposed. The proposed definition was applied to determine the number of PNRSV genetic strains in each of the plant samples and the complexity in defining genetic strains in multipartite genome viruses was explored.

## Introduction

RNA viruses are genetically heterogeneous and their replication mechanism results in mutants or variants that may differ genetically from the original virus [1]. A single virus isolate (representing a single specific sample of virus) does not consist of a single RNA sequence, rather, it consists of a population of related sequence variants, often referred to as ‘quasi-species’, which are generated due to the error-prone nature of the viral RNA-dependent RNA polymerase and rapid replication of virus genomes [2–4]. These populations of sequence variants are significant in defining strains that make up virus species and have been shown to have implications in the emergence of new and fitter virus strains that result from changing selection pressures [2] and could complicate their diagnosis. The occurrence of quasi-species, or sequence variants, compounds the already complex challenge of defining strains for viruses with multipartite genomes. Multiple combinations of genome components, inherited from different parental lineages, may lead to the selection and emergence of highly virulent strains and wide host range [5–7]. Equally, the polymorphisms could lead to an accumulation of less virulent strains that are more persistent in the population due to their lack of symptom expression and latent infection status.

RNA viruses have been widely characterised genetically using direct sequencing of either virus amplicons generated from RT-PCR or sequencing the molecular clones of individual RT-PCR amplicons [8–10]. Although direct sequencing of a population of RT-PCR amplicons may be informative, the output is a consensus sequence of variants present in a virus-infected sample, which limits the suitability of direct sequencing in virus population diversity studies. Until recently, cloning and sequencing of individual RT-PCR amplicons has been the alternative method used to direct sequencing to assess the genetic diversity of RNA virus populations. However, this approach is time consuming and labour intensive, thus limiting virus population surveys to a few clones that can be feasibly sequenced [11]. It is also likely that cloning only identifies the sequence variants occurring with high frequency and some infrequent, but potentially important, variants may be missed. This pool of infrequent variants may be a reservoir on which selection can occur and from which fitter and more pathogenic virus variants and strains can emerge [12].

Next Generation Sequencing (NGS) allows for rapid and deep viral sequence coverage that is an obvious improvement for the characterization of RNA viruses compared to cloning and sequencing of cDNA fragments [13, 14]. However, most applications of NGS, especially in plant virology, are designed for virus discovery and full genome sequencing, using de novo assembly and reference mapping tools to generate a virus consensus sequence output [13, 15, 16]. In addition, most sample preparation methods lead to high background levels of host sequences compared to virus sequences associated during NGS [17, 18]. The consensus sequence and high levels of non-viral sequence can limit NGS to the identification of frequently occurring virus sequence variants.

NGS of individual virus PCR amplicons offers an alternative approach to investigate diversity of virus populations, allowing targeted analysis of a specific region of the virus genome at greater depth of coverage [19, 20] and minimizes background host sequence interference during bioinformatics analysis. The high coverage, depth and targeted approach that NGS of PCR amplicons provides also makes it possible to pool multiple virus amplicons in a single sequence run. NGS of amplicons also detects populations of both high and low-occurring virus sequence variants [11]. Amplicon sequencing of viral sequence variants has largely been applied to human or animal viruses [11, 12, 21, 22]. The few studies on plant viruses have focused on viruses with monopartite genomes [23–25]. Less attention has been given to amplicon NGS of plant viruses with multipartite genomes and the subsequent analysis of observed sequence variants within each RNA component.

*Prunus necrotic ringspot virus* (PNRSV) (genus *Ilarvirus*) has a positive-sense, single-stranded tripartite RNA genome of 7,868 nucleotides (nt) in size, with each RNA component separately encapsidated with coat protein into icosahedral particles. RNA1 (3,332 nt) and RNA2 (1,943 nt) encodes for replicase proteins; and RNA3 (1,951 nt) encodes a movement protein (MP) and a coat protein (CP) [26, 27]. PNRSV has been traditionally grouped into three serotypes [28] that have more recently been confirmed based on phylogenetic analysis of PNRSV RNA3 CP sequences [29, 30]. These three PNRSV phylo-groups (PV32, PV96 and PE5) are based on RNA3 CP and are currently widely used for PNRSV isolate speciation [10, 31, 32]. However, these phylo-groups are only based on consensus sequences derived from cloning and/or direct Sanger sequencing of PNRSV isolates. It is unknown if low level variants exist within these phylo-groups and how these variants may contribute to PNRSV phylogeny. In addition, it is difficult to know if similar phylo-groupings occur across all the three RNA components of the PNRSV genome and how the combination of RNA variants within each component might represent groups of PNRSV genetic strains.

In this study, a broadly applicable approach of amplicon NGS and sequence analysis is described for virus genetic strain and diversity determination within and between virus-infected plant samples. The approach was successfully applied to PNRSV, which has a multipartite genome using RT-PCR amplicons from conserved regions of methyltransferase (MT), RNA dependent RNA polymerase (RdRp) and coat protein (CP) genes on RNA1, RNA2 and RNA3 respectively of PNRSV-infected *Prunus* trees (as determined by positive RT-PCR amplification with PNRSV-specific primers of RNA3). The sequence data generated were analysed using phylogenetic and identity-based methods to determine the relationship of the PNRSV amplicon sequence variants and from this analysis a definition of a PNRSV genetic strain is proposed.

## Materials and methods

### Plant material and total RNA extraction

Leaf tissue samples from 53 *Prunus* trees that had previously been found to be infected with PNRSV by RT-PCR test with PNRSV-specific primers of RNA3 [33], were collected in spring (2014–15) from five states in Australia through channels provided by the Almond Board of Australia and the Victorian state government (S1 Table). Total RNA was extracted from 0.3g leaf tissue (fresh weight) of each sample using the RNeasy**®** Plant Mini Kit (Qiagen) as described previously [33].

### RT-PCR amplification and amplicon purification

The SuperScript™ III One-Step RT-PCR System (Invitrogen) was used for the amplification of 218 bp, 387 bp and 455 bp segments of the methyltransferase (MT) gene on RNA1, the RNA dependent RNA polymerase (RdRp) gene on RNA2, and the coat protein (CP) gene on RNA3, respectively (Table 1). One step RT-PCR was conducted according to the manufacturer’s instructions except that the total reaction volume was 25 μl. Cycling conditions consisted of: a reverse transcription step at 50°C for 30 minutes; denaturation at 95°C for 15 minutes, followed by 35 cycles, denaturing at 95°C for 1 minute, annealing for 30 seconds at 56°C, 60°C and 54°C for MT, RdRP and CP primer pairs respectively, elongation at 72°C for 1 minute; and a final elongation step at 72°C for 10 minutes.

**Table 1:** Primers used for the RT-PCR amplification of partial methyltransferase (MT), RNA-dependent RNA polymerase (RdRp) and coat protein (CP) gene sequences of RNA1, RNA2 and RNA3 of the *Prunus necrotic ringspot virus* (PNRSV) genome respectively.

Primers	Primer sequence (5’-3’)	RNA (Gene Target) *	Amplicon length	Reference
PNRSV-MT-1	TCGAGCGATCTATTCCTAAG	RNA1 (MT)	218bp	This study
PNRSV-MT-2	ATCAATCACCGATTCTTCAG			
PNRSV-Rd-1	TTTTCACCGCTTTAGGTGCT	RNA2 (RdRp)	387bp	This study
PNRSV-Rd-2	GAACCTTCTTTCCCCCACTC			
PNRSV-C537	ACGCGCAAAAGTGTCGAAATCTAAA	RNA3 (CP)	455bp	[33]
PNRSV-H83	TGGTCCCACTCAGAGCTCAACAAAG			

*Gene targets encoding: MT = methyltransferase; RdRp = RNA-dependent RNA polymerase; CP = Coat Protein.

To confirm successful amplification of the MT, RdRp and CP genes segments in each of the 53 samples, 8 μl of each of the RT-PCR reactions were run on a 1% agarose gel and stained with SYBR® Safe DNA gel stain (Invitrogen) for visualization. The remaining 17 μl of each the 159 PNRSV RT-PCR amplicons were purified using the Promega Wizard® PCR clean-up kit (Promega) according to the manufacturer’s instructions.

### Library preparation for next generation sequencing

Twenty μl of each of the 159 purified amplicons were used to prepare the NGS amplicon libraries. For Illumina dual indexing, adapter mpxPE2 consisting of unique barcodes for each amplicon were ligated to the 3’-terminus to incorporate the sequencing primer site, while an adaptor containing one of 8 indexing sequences 5 bp in length (mpxPE1), was ligated to the 5’-terminus of each of the amplicons using the NEBNext® T4 ligase (New England BioLabs) in separate 30 µl reactions. The AMPure XP® bead solution (Beckman Coulter) was used to purify the adapter-ligated amplicons prior to PCR enrichment.

For PCR enrichment, 6 μl of each of the adapter-ligated amplicons were used in separate 50 μl reactions each containing 4 μl of in-house multiplex PE barcode primer mix (2.5 μM) that was made up of the 8 mpxPE1 primers and mpxPE2 primers which were unique for each of the 159 amplicons and 40 μl Phusion® high fidelity PCR mastermix (New England BioLabs). PCR cycling conditions consisted of: one cycle at 98°C for 30 seconds, 15 cycles at 98°C for 10 seconds, 65°C for 30 seconds, 72°C for 30 seconds; and a final extension step at 72°C for 5 minutes. After the PCR, excess primers and any primer dimer present were removed using Ampure XP® system (Beckman Coulter) according to the manufacturer’s instructions and the final amplicon libraries eluted in 20 μl of RNAse-free water.

The size distribution and concentration of the amplicon libraries were determined using the 2200 TapeStation® system (Agilent technologies) and Qubit® Fluorometer 2.0 (Invitrogen) respectively. The resulting quantification values were used to pool the amplicon libraries at equal concentration. The pooled library was diluted to 4 nM, denatured, and diluted to a final concentration of 12 pM [34]. The amplicon library was sequenced using the Illumina MiSeq with a paired read length of 301 base pairs. The sequence read data for this study have been submitted to the NCBI Sequence Read Archive (SRA) database under the Bioproject accession PRJNA326695 and SRA study accession SRP077034.

### Amplicon sequence reads pre-processing

The sequence reads were quality filtered, the adapter sequences were removed, and the sequence data subjected to read pair validation using Trim Galore! (version 0.4.0), with a quality score of >20 and minimum read length of 200 nt. [35]. The quality trimmed reads were then paired using PEAR (version 0.9.4), with default parameters [36] to generate paired-end reads.

To ensure amplicon sequence reads from each PNRSV RNA gene segment had similar length and similar sequence start and stop, the paired reads in fastq format were first converted into FASTA format using fastq_to_fasta function of FASTX-Toolkit [37]. A local BLAST analysis with default settings using BLASTn (version 2.2.18) [38] was done to ensure all the paired amplicon sequence reads were in the correct forward orientation. Amplicon sequence reads that had a reverse orientation were reversed and complemented using fastx_reverse_complement function of FASTX-Toolkit [37]. The amplicon reads were then aligned using Muscle (version 3.8.31) [39] with default parameters. The overlapping alignment coverage for each RNA amplicon read was identified and Cutadapt (version 1.4.1) [40] was used to trim each RNA amplicon read as follows: 10 nt from 5’ end and 8 nt from the 3’ end of RNA1; 14 nt from the 5’ end and 23 nt from the 3’ end of RNA2; and 24 nt from the 5’ end and 31 nt from the 3’ end of RNA3. The resulting length of all amplicons reads for each of RNA1, RNA2 and RNA3 were 200 nt, 350 nt and 400 nt respectively; shorter reads in each set of amplicons were discarded.

The read depth for each of the MT (RNA1), RdRP (RNA2) and CP (RNA3) amplicons in of each of the 53 plant samples was calculated as the percentage fraction of number of reads of each amplicon to a combined total number of trimmed reads for all three amplicons.

### Amplicon read clustering

Groups or clusters of unique sequence variants for each gene segment and their frequency were determined using Usearch (version 7.0.1090) [41]. Prior to analysis, the Illumina barcode read labels on each of the 159 amplicons were replaced with the name of the tree samples and RNA amplicon component using the Usearch fastq_strip_barcode_relabel.py python script. The Uclust function in the Usearch program was then used to cluster the relabeled amplicon reads at 100% identity/similarity and to determine the proportion of reads assigned to each cluster.

The resulting clusters were then sorted in descending order according to the proportion of reads represented using the Usearch “sortbysize” function. The clusters including single reads that did not have 100% identity to any other read were translated into amino acid sequences using Emboss Transeq (version 6.6.0) with default parameters [42] to identify the proportion of non-coding sequence clusters. The encoding amplicon cluster sequences were then translated back into nucleotide sequences using Emboss Backtranseq (version 6.6.0) [42] with default parameters.

### Amplicon sequencing error detection control and filtering

Three transcribed RNA (trRNA) controls were produced from a single *Prunus* isolate cloned PNRSV RNA3 RT-PCR amplicon to assess the rate of sequence error introduced during RT-PCR and sequencing. To produce the controls, three RNA3 amplicons were cloned using the pGEM^®^-T Easy vector system (Promega). Three plasmids containing inserts of the expected size were selected and verified by DNA sequencing (BigDye v3.1 chemistry sequencing kit, Applied Biosystems). The plasmids were linearized using the *SalI* restriction enzyme (Promega) and transcribed into RNA using a MEGAScript^®^ T7 Kit (Invitrogen), as recommended by the manufacturer.

DNA was removed from the trRNA using a DNA-*free*™ kit (Invitrogen) according to the manufacturers’ instructions. PCR with PNRSV RNA3 primers (Table 1) was used to detect any remaining DNA in the DNase treated 20 μl trRNA reaction volume using Platinum™ *Taq* DNA Polymerase kit (Invitrogen) according to the manufacturer's instructions. PCR cycling conditions were: 1 cycle of 94°C for 2 minutes followed by 35 cycles of 94°C for 1 minute, 54°C for 30 seconds, and 72°C for 1 minute; with a final extension of 72°C for 10 minutes. The DNase treatment was repeated as many times as necessary until DNA was not detected in the trRNA.

The three trRNA3 products were reverse transcribed and amplified using SuperScript™ III One-Step RT-PCR System (Invitrogen) and RNA3 primers (Table 1) as previously described. Separate libraries were prepared for each of the resulting amplicons and these were sequenced using the Illumina MiSeq with a paired read length of 301 base pairs as previously described. The resulting sequence reads were pre-processed and clustered at 100% identity as described previously.

The following process was used to determine the proportion (%) of error-associated reads and the error rate (%) occurring during RT-PCR and amplicon NGS: clusters of amplicon sequences for each trRNA control were compared to the original sequence of each clone; clusters that were not 100% identical to the original cloned PCR product were presumed to be error-associated; these error-associated clusters were also translated into amino acids using Emboss Transeq (version 6.6.0) with default parameters [42] to identify the proportion of non-coding clusters; the total number of amplicon sequencing reads in the error-associated clusters was divided by the total number of reads in each triplicate amplicon control to calculate the average proportion (%) of error-associated with RT-PCR and amplicon NGS; the NGS amplicon error rate (%) was calculated as the average of the proportion (%) of total nucleotide bases in the error-associated reads that were different to the source clone’s sequence compared to the total nucleotide bases in each triplicate amplicon control.

It was assumed that this proportion of error was the same for the NGS data for each of the 159 amplicons. Therefore, a range of cluster sequences containing the same proportion of error-associated reads in ascending order starting from singletons and non-coding variants clusters were removed from the 159 amplicon samples data. The remaining filtered sequence variants from the 159 amplicon samples was used for phylogenetic and sequence identity analysis.

### Phylogenetic and sequence identity analysis

Prior to phylogenetic and sequence identity analysis, sequences of the PNRSV type reference and phylo-groups sequences (AF278534, AF278535, U57046, Y07568, S78312, L38823; S2 Table) were retrieved from GenBank and trimmed according to each corresponding region of the genome that was amplified from each of the 53 *Prunus* tree samples and subsequently analysed in this study.

Nucleotide sequence from the type isolates and each of the filtered amplicon clusters, from all samples and for each RNA segment were aligned using Muscle (version 3.8.31) [39]. A maximum likelihood phylogenetic tree was constructed in RAxML (version 8.0.19) [43] using GTRGAMMA model with 1000 bootstrap replicates. The resulting trees were visualized in FigTree version 1.4.2 [44] and branches that had less than 90% bootstrap support were collapsed. Sequence identity analysis using the sequence demarcation tool (SDT) (version 1.2) [45] was carried out on the aligned amplicon clusters of each RNA and also on each of the individual amplicon clusters.

### Amplicon cluster intra-host relationships

Haplotype networks were created in order to visualize the genetic variation and evolutionary relationship of amplicon sequence variant clusters for each of PNRSV RNA1, RNA2 and RNA3 at the intra-host level. The haplotype networks were constructed using Median Joining (MJ) algorithm [46] and visualized using PopART software (http://popart.otago.ac.nz).

### Amplicon sequence variant calling and annotation

Each of the error-filtered RNA amplicon sequence variants were mapped to their respective PNRSV type reference (S2 Table) using the BWA (version 0.6.2) with default parameters [47]. The generated mapping SAM file was converted to BAM file format using Samtools (version 0.1.18) with default parameters [48]. Sequence mutations were then detected using Freebayes (version 1.0.2) with default parameters[49]. The resulting variant calling format (VCF) files were annotated using SnpEff (version 4.2) [50], to determine the functional significance of mutations identified.

## Results

### Next generation sequencing data of PNRSV amplicons

The total number of raw reads was 3,431,678 when the NGS amplicon data for PNRSV MT, RdRp and CP gene segments of RNA1, RNA2 and RNA3 respectively, which were amplified from all 53 plant samples, were combined. The total number of reads for MT, RdRp and CP amplicons were 1,571,434, 541,757 and 1,318,487 respectively. After quality trimming there was an overall total number of 3,140,735 reads used for analysis and the read numbers for the MT, RdRp and CP amplicons were reduced to 1,449,457, 468,106 and 1,223,172 respectively (Table 2). The read depth for PNRSV MT, RdRp and CP amplicons in each of the 53 plant samples, when compared to the overall total number of quality trimmed reads, ranged from 0.55–1.19%, 0.2–0.52% and 0.45–1.02% for MT, RdRp and CP gene segments amplified from RNA1, RNA2 and RNA3 respectively (S3 Table).

**Table 2:** The number of reads generated from the next generation sequence of the amplicons derived from PNRSV RNA1, 2 and 3 and the number of sequence variants from cluster analysis.

	RNA1 (Partial MT gene: 200bp)			RNA2 (Partial RdRp gene: 350bp)			RNA3 (Partial CP gene: 400bp)		
Plant ID	No. of reads after trimming	No. of sequence variants	No. of sequence variants after filter	No. of reads after trimming	No. of sequence variants	No. of sequence variants after filter	No. of reads after trimming	No. of sequence variants	No. of sequence variants after filter
11TAS	30904	1025	91	9446	2848	23	21244	5688	71
12TAS	32602	1257	112	8737	2539	49	19821	4618	68
13TAS	22536	874	69	8486	2704	33	21934	7569	110
14TAS	17316	786	71	10880	3409	87	21319	6283	91
15TAS	21600	853	75	8362	1946	28	25592	6461	122
16TAS	36173	1658	107	9125	2089	30	23585	5311	67
17TAS	27850	996	74	8648	2838	34	21964	7011	102
1SA	23154	718	67	7052	1202	21	22572	6917	137
1TAS	30199	1186	98	9577	2242	24	25797	7154	117
2TAS	37383	1263	143	10228	1553	28	23550	8803	128
2VIC	23824	907	81	8964	2180	31	31526	11030	138
3VIC	29200	1092	117	8295	1978	19	31999	7890	100
4TAS	17054	811	65	6812	1718	37	19228	5755	63
5SA	24453	647	59	8347	2303	33	24649	6997	82
5TAS	29755	1636	123	7269	2136	21	29469	8809	145
6VIC	25089	1005	100	9533	2758	47	28395	7547	123
Cst	17553	950	62	8859	2586	26	26084	7270	104
K10	27873	754	55	8768	2423	38	29295	7841	127
K16	34786	1092	101	9535	2808	25	25087	6257	103
K17	34353	1291	114	11001	3617	61	19864	6408	130
K56	29974	1114	96	9859	3039	73	24397	7032	135
K57	31863	1164	74	9467	2880	50	21062	4103	62
K64	29699	1843	173	6991	2168	35	26794	8091	161
K72	35987	834	81	7386	2171	42	20951	5142	78
K75	32421	916	94	10334	2939	33	19704	5393	65
K77	28754	1319	141	6413	2152	24	32043	10877	183
M10	26806	1428	155	8475	2622	31	22674	6034	100
M12	26023	1607	174	10290	3218	49	24056	5778	65
M16	19506	613	41	9394	2721	43	22546	5827	96
M17	35484	1898	223	8585	2581	30	27442	7365	114
M18	32387	921	72	7490	2357	42	18261	4150	53
M19	32439	1507	169	16422	8637	140	24284	5945	70
M26	19751	725	61	9407	2790	53	22326	7858	139
M28	24887	835	83	7555	2232	24	19399	6195	111
M30	25038	598	53	10348	3217	39	24746	5740	80
M31	28125	1131	92	10201	2776	41	15976	3358	57
M32	21901	553	34	7186	2178	29	23941	7594	113
M33	22916	1056	109	7956	2348	37	21569	6309	122
M5	25003	789	74	8642	2650	25	17777	4360	68
M6	32807	1235	92	7611	2272	44	20664	5869	81
M8	20668	690	60	9368	3172	63	21148	6881	101
M9	32606	1060	111	6240	1713	22	14257	3990	42
NS10	33531	1300	136	7679	2217	37	19800	5814	69
NS1	19885	617	53	7687	2216	34	17368	3520	41
NS2	22193	797	74	9332	3024	41	21271	6975	121
NS4	32160	797	99	6890	1384	23	30513	11095	202
NS9	31820	969	131	8737	2726	37	20627	5069	88
Pch4	21550	634	43	8284	2541	33	21983	6756	105
Q15	29111	1150	152	9324	2908	40	23576	6674	106
Q16	22590	890	32	7668	2366	35	21359	5258	115
Q1	23303	704	45	8422	2604	52	17260	6606	119
Q6	25320	995	94	11147	3664	60	25579	7252	155
Q7	29292	1217	135	9392	2756	27	24845	6031	140
**Average**	**27348**	**1032**	**95**	**8832**	**2625**	**39**	**23079**	**6539**	**104**

The table contains the number of sequence reads obtained from next generation sequencing of RT-PCR amplicons from conserved regions of methyltransferase (MT), RNA dependent RNA polymerase (RdRp) and coat protein (CP) genes on RNA1, RNA2 and RNA3 respectively of the 53 PNRSV-infected *Prunus* samples and the resulting number of sequence variant from the sequence reads clustering at 100% identity and the final number of error-filtered sequence variants

### Amplicon read clustering

Clustering of the trimmed amplicon sequence reads at 100% identity resulted in unique sequence variants averaging at 1,032, 2,625 and 6,539 variants per sample for MT, RdRp and CP gene segments respectively, in each of the 53 plant samples (Table 2). Singletons accounted for an average of 2%, 25% and 24% of total reads of MT, RdRp and CP gene variants respectively (S5, S6 and S7 Tables) and 80% of these reads resulted in non-coding amino acid sequence. On translation of all of the sequence variants, excluding singletons, into amino acid sequence, there were an average of 50 and 44 non-coding variant clusters for each of MT, and CP gene segments respectively across the 53 plants samples and more than 95% of these non-coding clusters had 2–10 reads (S6 and S8 Tables). Only three plant samples had non-coding variant clusters (57, 69 and 42 clusters) of RdRp gene segment and all of these were also clusters comprised of 1–10 reads (S7 Table).

### Amplicon sequencing error detection control and filter

Sequencing and analysis of PCR amplicons from transcribed RNA (trRNA) error detection controls showed that the most abundant sequence in all analyses (i.e. the sequence with the highest frequency number of reads) in each triplicate control was 100% identical to their original source clone’s sequence. It also revealed a high proportion of singletons averaging 29% of total reads and non-coding clusters averaging 20% of total clusters. This indicated that low-occurring variants that includes singletons and non-coding variants are error-associated. These error-associated reads averaged 37% of the total reads generated in the error detection control (S4 Table) with an average amplicon error rate of 0.6% (S5 Table).

Based on the determined proportion of amplicon error-associated reads, 37% of total reads in ascending order, starting from singletons, were identified to occur mainly in low level variants with less than 10 reads in the 159 amplicons generated from the plant samples. Therefore, all variant sequences made up of less than 10 reads and non-coding variant sequences were removed from the data. A total of 1,034,572 (32%) of amplicon sequence reads were removed from the combined total of 3,140,735 quality trimmed and pre-processed reads. This resulted in a decrease of the average number of sequence variants per plant sample from 1,032 to 95 variants for the MT gene segment; 2,625 to 39 variants for the RdRp gene segment and 6,539 to 104 variants per sample for the CP gene segment (Table 2).

### Phylogenetic analysis and sequence identity analysis

**Fig 1:**
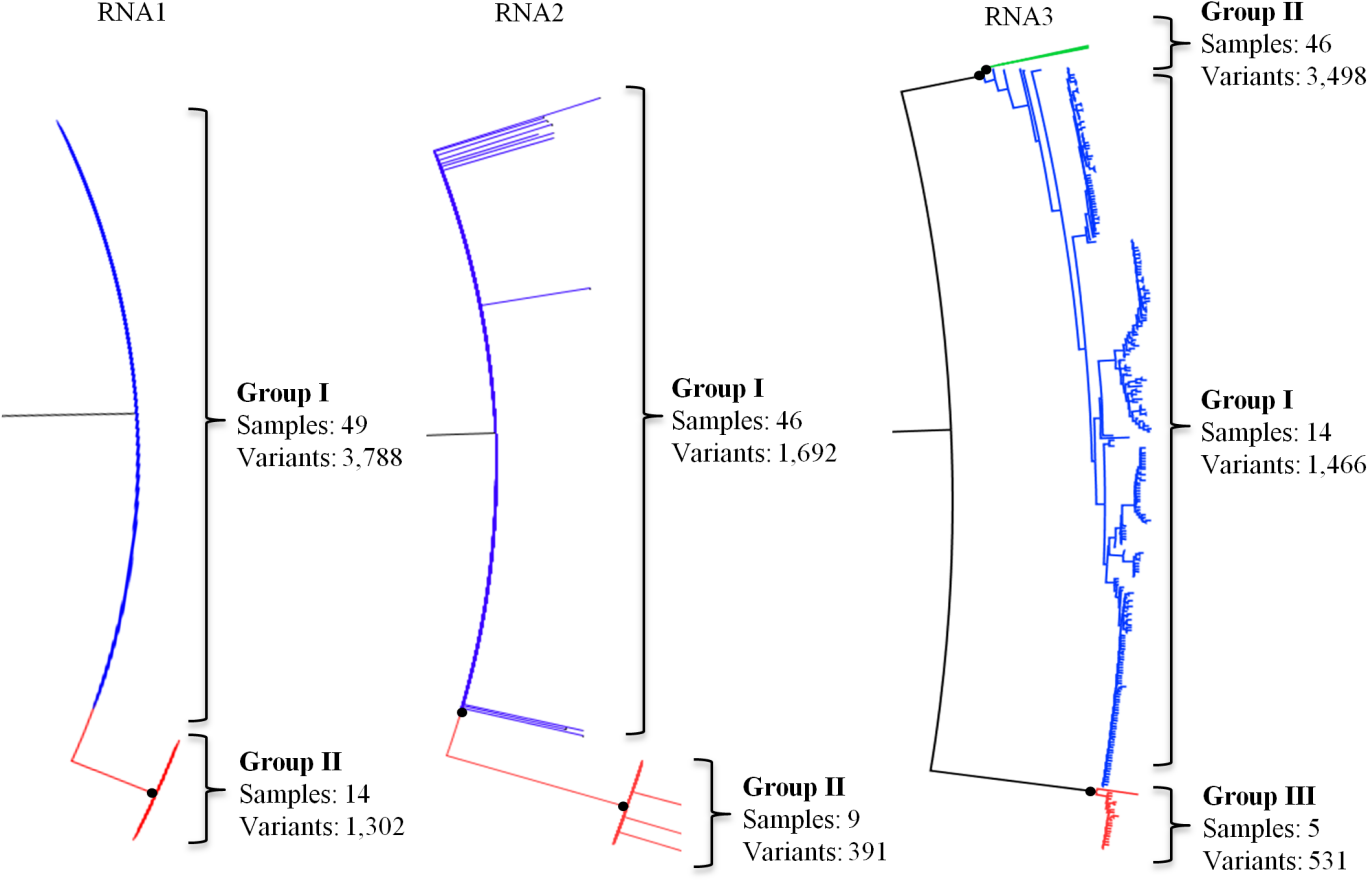
Maximum likelihood relationship phylogeny obtained from alignment of 5,040, 2,083 and 5,486 amplicon sequence variants of methyl transferase (MT), RNA dependent RNA polymerase (RdRp) and coat protein (CP) gene segments on PNRSV RNA1, RNA2 and RNA3 respectively. The alignments were generated using Muscle version 3.8.31 and the phylogenetic trees constructed with RAxML version 8.0.19 using GTRGAMMA model with 1000 bootstrap replicates. The trees were visualized in FigTree version 1.4.2 with branches having less than 90% bootstrap support collapsed and are indicated by the black circles. Sequence variant labels were removed for ease of presentation and major variant groups colour coded with phylo-group II on RNA3 (shown in green colour), which had the largest number of variants, collapsed for ease of presentation. The number of samples and number of sequence variants forming each major group of each RNA are shown.

Phylogenetic analysis of the error-filtered MT, RdRp and CP amplicon sequence variants of RNA1, RNA2 and RNA3 respectively from all 53 plant samples and the published PNRSV type reference and type phylo-groups provided >95% bootstrap support for two phylogenetic groups for each of RNA1 and RNA2 and three phylogenetic groups for RNA3 (Fig 1). In this study the phylo-groups PV32-I, PV96-II and PE5-III [30, 51] were referred to as PG1, PGII and PGIII respectively and the phylogenetic groupings for both RNA1 and RNA2 were designated as PGI and PGII in accordance with this naming criteria. The largest phylo-groups for each PNRSV RNA segment were RNA1 PGI, RNA2 PGI and RNA3 PGII having 3788, 1692 and 3498 sequence variants respectively and occurred in a larger proportion of plant samples compared to the other phylo-groups. RNA1 PGI occurred in 49/53 plants samples, RNA2 PGI and RNA3 PGII each occurred in 46/53 plant samples (Fig 1).

Seventeen of the 53 plant samples had amplicon sequence variants occurring in two or three phylogenetic groups of at least one of RNA1, RNA2 and/or RNA3 segments and 36/53 plant samples had amplicon sequence variants belonging to a single phylogenetic group for each RNA segment (Table 3). The most frequently occurring combination of phylo-groups was RNA1 PGI, RNA2 PGI and RNA3 PGII (34/53 plant samples; Table 3).

**Table 3:** The variant phylo-groups identified from phylogenetic and identity analysis of amplicon variant sequences of the 53 *Prunus necrotic ringspot virus* (PNRSV) samples, the % clusters identity cut-off and the resulting minimum number of variant groups in each amplicon sample RNA.

	Variant phylo-groups			Variants % identity cut-off			Minimum number of variant groups		
Sample ID	RNA1	RNA2	RNA3	RNA1	RNA2	RNA3	RNA1	RNA2	RNA3
11TAS	I	I	II	98%	98%	98%	1	1	1
12TAS	II	II	I & II	99%	98%	97% & 97%	1	1	2
13TAS	I	I	II	98%	98%	98%	1	1	1
14TAS	I	I	II	98%	98%	99%	1	1	1
15TAS	I	I	II	98%	99%	99%	1	1	1
16TAS	I	I	II	97%	98%	97%	1	1	1
17TAS	I	I	I, II & III	98%	98%	99%, 97% & 97%	1	1	3
1SA	I	I	II	98%	98%	98%	1	1	1
1TAS	I	I	II	98%	98%	97%	1	1	1
2TAS	I	I	II	98%	99%	97%	1	1	1
2VIC	I	I	II	98%	98%	98%	1	1	1
3VIC	I	I	II	98%	98%	98%	1	1	1
4TAS	I & II	II	I	97% & 98%	99%	99%	2	1	1
5SA	I	I	II	98%	99%	97%	1	1	1
5TAS	I & II	I	I & II	98% & 98%	99%	98% & 97%	2	1	2
6VIC	I	I	II	98%	98%	98%	1	1	1
Cst	I	I	II	98%	98%	98%	1	1	1
K10	I	I	II	98%	98%	98%	1	1	1
K16	I	I	II	98%	98%	97%	1	1	1
K17	I & II	I	I & II	97% & 97%	98%	97% & 98%	2	1	2
K56	I	I	II	98%	99%	98%	1	1	1
K57	I	I	I & II	98%	98%	99% & 98%	1	1	2
K64	II	I & II	I & III	98%	97% & 98%	97% & 98%	1	2	2
K72	I	I	II	98%	99%	97%	1	1	1
K75	I & II	II	I	99% & 98%	98%	98%	2	1	1
K77	II	II	I & II	98%	99%	97% & 99%	1	1	2
M10	I	I	II	98%	98%	98%	1	1	1
M12	I	I	II & III	98%	98%	97% & 97%	1	1	2
M16	I	I	II	98%	98%	97%	1	1	1
M17	I	I	II	98%	98%	98%	1	1	1
M18	II	II	I	98%	98%	97%	1	1	1
M19	I & II	I & II	I & II	97% & 97%	98% & 99%	97% & 99%	2	2	2
M22	I	I	II	98%	99%	97%	1	1	1
M26	I	I	II	98%	99%	97%	1	1	1
M28	I	I	II	98%	99%	99%	1	1	1
M30	I	I	II & III	98%	98%	98% & 97%	1	1	2
M31	I & II	I	II	97% & 97%	98%	97%	2	1	1
M32	I	I	II	98%	98%	98%	1	1	1
M33	I	I	II	98%	98%	99%	1	1	1
M5	I	I	II	98%	99%	97%	1	1	1
M6	I	I	II	99%	99%	98%	1	1	1
M8	I	I	II	98%	99%	98%	1	1	1
M9	I	I	II	98%	99%	97%	1	1	1
NS10	I & II	I	I & II	99% & 97%	98%	97% & 98%	2	1	2
NS1	I	I	II	98%	98%	98%	1	1	1
NS2	I	I	II	98%	98%	97%	1	1	1
NS4	I	I	I	97%	98%	97%	1	1	1
NS9	I & II	II	I	98% & 99%	99%	97%	2	1	1
Q15	I & II	II	I, II & III	97% & 97%	98%	99%, 97% & 97%	2	1	3
Q16	I	I	II	98%	99%	98%	1	1	1
Q1	I	I	II	98%	99%	97%	1	1	1
Q6	I & II	I	II	98% & 98%	98%	98%	2	1	1
Q7	I	I	II	98%	98%	98%	1	1	1

SDT identity analysis was used to determine the identity demarcation of sequence variants in each of the 159 amplicons and the phylogenetic groups. Variants in each of the 159 amplicons had an identity cut-off of more than 97% with the exception of amplicons with variants occurring in multiple phylogenetic groups (Table 3). SDT identity analysis of amplicon sequence variants of each RNA showed similar groupings as observed in the phylogenetic analysis. Variants occurring within the same phylo-group for each of RNA1, RNA2 and RNA3 shared more than 97% similarity (Table 3). The similarity of variants between phylo-groups for each of RNA1, RNA2 and RNA3 was less than 97% with some variants sharing as little as 84% similarity between phylo-groups (Table 3).

### Amplicon sequence variants intra-host relationships

**Fig 2:**
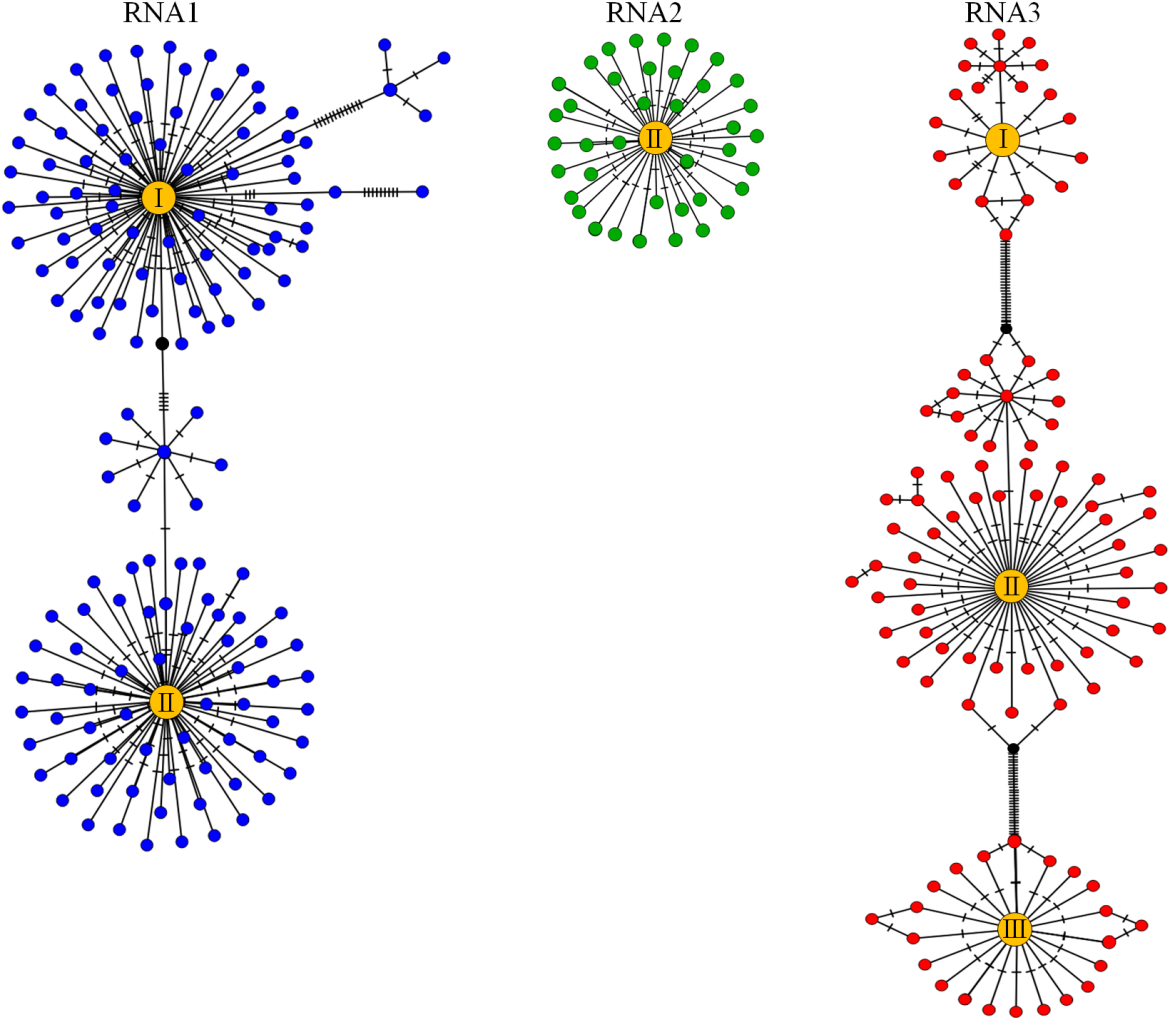
Median-joining haplotype network showing *Prunus necrotic ringspot virus* (PNRSV) intra-specific diversity of RNA1, RNA2 and RNA3 sequence variants in sample Q15. Each of the blue, green and orange circles represents a PNRSV methyl transferase (MT), RNA dependent RNA polymerase (RdRp) and coat protein (CP) amplicon sequence variant of RNA1, RNA2 and RNA3 respectively. The yellow circles represent the largest sequence variants (variant made up of highest copy number of reads) of each phylo-group (labeled I, II or III) of RNA1, RNA2 and RNA3 present in sample Q15. The hatch mark indicates the number of mutations separating the haplotypes and the black circles (median vectors) are hypothetical missing intermediates connecting the haplotype groups. Multiple median vectors connecting phylo-groups were collapsed for ease of presentation.

Median-joining haplotype networks were inferred to show the genetic and evolutionary relationship within each MT, RdRp and CP amplicon sequence variant in a sample. As an example, the haplotype network for the MT, RdRp and CP amplicon sequences of PNRSV of sample Q15 is shown in Fig 2 and the results indicate that there was a clear separation of variant clusters based on their RNA1, RNA2 and RNA3 phylo-groups. This trend was observed in the haplotype networks of all 53 amplicon samples (data not shown). In each RNA haplotype network, low-frequency sequence variants (variants made up of low copy number of reads) appeared to diverge in a star-burst pattern from the largest sequence variant (variants made up of highest copy number of reads) The multiple phylo-groups of RNA1 and RNA3 occurring in sample Q15 appeared as distinct populations separated by a series of nucleotide base changes in the haplotype networks.

### Amplicon sequence variant calling and annotation

Variant calling on each of the RNA amplicon error-filtered sequence variants identified a total of 389, 211 and 682 nucleotide polymorphic sites on the amplified MT, RdRP and CP of RNA1, RNA2 and RNA3 respectively (S9 Table). The analysis showed that 387 of the 389 changes observed in RNA1 were synonymous mutations (S9 Table) with only one sample having 2 non-synonymous mutations that resulted in a missense substitution (S10 Table). In contrast, the majority of nucleotide changes observed in RNA2 and RNA3 (199 of 211 and 536 of 682 respectively) were non-synonymous mutations (S9 Table). All RNA2 and RNA3 non-synonymous mutations resulted in missense substitutions. In addition, all RNA3 amplicon samples belonging to PGII and III in 45 of the plant 53 samples each had a single hexa-nucleotide in-frame deletion mutation (S10 Table).

## Discussion

This study describes a novel use of amplicon NGS to investigate the diversity of a tripartite genome virus, PNRSV, by focusing on conserved regions within the MT, RdRp and CP genes of each RNA component of the genome. The high sequence read depth associated with amplicon NGS allowed for an improved estimation of PNRSV diversity within and between plant samples when compared to other methods used for estimating virus diversity.

This is the first in depth study of the genetic diversity of PNRSV RNA components 1 and 2. It provides evidence for two distinct PNRSV phylo-groupings for each of RNA1 and RNA2, which were observed for similar gene regions of RNA1 and RNA2 of the *Bromoviridae* family members *Alfalfa mosaic virus* (AMV) and *Cucumber mosaic virus* (CMV; genus *Cucumovirus*) [52, 53]. Phylogenetic analysis of CP sequence variants from the 53 plant samples diverged into the three already defined PNRSV RNA3 phylo-groups [26, 27, 30, 32], even though a smaller portion of the genome was used. The phylogenetic groups identified for RNA1, RNA2 and RNA3 of PNRSV in the 53 plant samples were further confirmed by pairwise sequence identity comparison of sequence variants within each PNRSV amplicon which had a ≥ 97% sequence identity. Therefore, an identity of 97% was determined as the demarcation threshold for each of the PNRSV RNA phylo-groups occurring within an amplicon sample.

The haplotype analysis also indicated that each phylo-group could consist of distinct clades of variants for each of the RNA1, RNA2 and RNA3 sequence variants within a plant sample and these were formed by smaller sequence variant clusters that were genetically connected but diverging from the largest sequence variant cluster (Fig 2). The occurrence of distinct multiple phylo-groups of an RNA component and distinct clades of variants within a phylo-group in a single plant (eg. Sample Q15; Fig 2) may indicate different infection events or infection at the same time by distinct RNA phylo-group variants or strains.

The PNRSV phylogenetic groups observed in this study were supported by the sequence identity analysis and haplotype networks, which indicates that these variant clusters within each of PNRSV MT, RdRp and CP amplicons represent a functional unit that is important in defining PNRSV species taxonomy. Virus species have widely been defined as a polythetic class whose members share a consensus group of properties but not all share a single common property [54–56]. These properties may include genome, biological and serological properties [57], and the variation in some of these properties among members of a virus species may be the differentiating element of species into variants. These variants of a virus species, can be further defined as strains if they are a genetically stable collection of variants that differ from the type variant in stable biological, serological or molecular characteristics [58–60].

In context to this study, phylogenetic analysis differentiated the PNRSV amplicon sequence variants in each RNA component into phylo-groups which represent distinct taxonomic units of each RNA component that may define a PNRSV genetic strain. We therefore propose the following definition of a PNRSV genetic strain based on our PNRSV amplicon sequence analysis: *A genetic strain of PNRSV in a biological isolate (plant) must comprise of at least one variant of each RNA component that encodes the expected open reading frame (ORF); and may include sequence variants that are ≥97% similar*. There is no formal taxonomic definition below the species level by ICTV for any plant virus and it may be possible to extend this proposed virus genetic strain definition to other species of both monopartite and multipartite genome viruses where functional proteins encoded by RNA component/s are required for a virus infection. However, like the demarcation criteria between virus species [61], the proposed 97% demarcation threshold assigned to PNRSV in this study may not hold for strains of other virus species and would need to be determined on a case by case basis.

In this study, the majority of plant samples (36 of 53) had variants of RNA1, RNA2 and RNA3 belonging to a single phylo-group and therefore could be described as having a single genetic strain of PNRSV. Based on the different possible combination of the phylo-groups for RNA1, RNA2 and RNA3 these 36 samples represent three PNRSV genetic strains. The most common PNRSV genetic strain consisting of single phylogenetic groups of each RNA component occurred in 34 samples and had the combination of RNA1 PGI, RNA2 PGI and RNA3 PGII [30]. The two remaining genetic strains each occurred in a single plant sample and consisted of RNA1 PGI, RNA2 PGI and RNA3 PGI and RNA1 PGII, RNA2 PGII and RNA3 PGI.

The existence of variants occurring in multiple phylo-groups for RNA1, RNA2 and/or RNA3 observed in 17 plant samples complicates the definition of a genetic strain for a multipartite virus and the determination of the number of PNRSV genetic strains present in a biological sample. The RNA components of a single-stranded RNA virus with a multipartite genome are frequently encapsidated separately allowing for pseudo-recombination or reassortment between the genome component variants [62, 63]. This leads to a complexity that presents a challenge in accurately determining the number of genetic strains of a multipartite genome virus present in a plant. For example, tree sample M19 had two phylo-groups in each of RNA1, RNA2 and RNA3, which could either mean M19 is infected with two PNRSV genetic strains or may be up to eight (2^3^) PNRSV genetic strains depending upon how the RNA components function together. Conversely in tree sample Q15 (Fig 2) there are two phylo-groups of RNA1 and RNA3 and one phylo-group of RNA2 suggesting that Q15 could either have 2 or 4 genetic strains of PNRSV depending on the different combinations the RNA components.

With this genetic complexity in mind, in this study each plant sample represents a biological isolate that may consist of one or more PNRSV genetic strains which are defined by the PNRSV RNA1, RNA2 and RNA 3 phylo-groups that were present. However, there was no association between PNRSV strains or specific RNA1, RNA2 or RNA3 phylogroups and host geographical origin or host specificity which supports similar observations of previous phylogenetic studies of PNRSV RNA3 [30, 64]. In most samples, symptoms specific to a PNRSV infection were not observed but symptom expression could have been affected by the time of year that the sample was taken as well as the specific *Prunus* species or variety that was sampled.

The raw and clustered filtered NGS data showed that each of the PNRSV RNA2 genome components occurred with lower frequency compared to RNA1 and 3 amplicon sequence reads. Similarly, infection studies with the related tripartite genome virus, *Alfalfa mosaic virus* (AMV; genus *Alfamovirus*, family *Bromoviridae*) also showed that RNA2 occurred at a lower frequency than the other RNA genome segments [65]. The lower number of RdRp amplicon sequence variants is likely due to the highly conserved nature of the RdRp gene, which encodes several motifs necessary for replication [66, 67]. However, the majority of nucleotide changes observed in RdRp and CP amplicon sequences were non-synonymous mutations which resulted in different amino acid sequence compared to the type isolate reference sequence [64, 68]. RdRp non-synonymous mutations were mis-sense substitutions and the biological significance of these amino acid sequence changes is not known.

CP amplicons of all 53 samples had mis-sense mutations and CP amplicons of 45 of these samples which belong to phylo-groups PGII and PGIII also had a single hexa-nucleotide in-frame deletion mutation. This six nucleotide deletion resulted in the deletion of two amino acids (Asp-Arg) when compared to the type reference [64] and has been reported previously as a characteristic feature of PNRSV CP PGII and III [30, 64]. The biological significance of this deletion is not known, but it does serve as a PNRSV CP PGII- and III-specific feature that can be used to discriminate against PGI.

Regardless of the high level of sequence diversity at the nucleic acid level, all MT sequence variants, with the exception of two variants in one sample, had synonymous mutations that resulted in no change at the amino acid level. This high level of PNRSV methyltransferase amino acid sequence conservation could be associated with the importance of the MT gene in PNRSV replication [67, 69], but might also be associated with less observed diversity due to the short RNA1 amplicon size (200 bp) used for this analysis.

The PNRSV amplicon NGS data from this study represent the minimum number of sequence variants within a virus isolate from a single sample of a tree at one time point. A high level of sequence diversity was observed across all the samples using NGS amplicon sequencing, with a total number of 5040, 2083 and 5486 sequence variants observed for MT, RdRp and CP amplicons respectively. This high level of diversity represents only a small portion of each RNA component and it is likely that a greater level of sequence diversity would be observed if the whole PNRSV genome sequence was analysed. Furthermore, the distribution of virus variant populations in a plant host can vary according to the plant tissue that was sampled [70, 71] and in this study, variant detection may be biased towards the leaf tissue that was used.

Errors can occur during PCR and amplicon NGS which may inflate diversity [72–75]. Several different approaches to remove amplicon NGS errors that use statistical software have been reported [76–80]. Despite the usefulness of these error correction methods, Schirmer et al. [81] found that various experimental factors have a major influence on error patterns of sequence data. Therefore a stringent internal error detection control and filtering process was specifically designed for the experimental factors in this study to minimise the occurrence of sequences with errors so that they do not have a significant impact on the outcome of the bioinformatics analyses. The PNRSV amplicon sequencing error rate of 0.6% determined in this study was within the 97% amplicon sequence identity cut-off observed within PNRSV amplicon sequence variants present in each RNA phylo-grouping. Therefore, any error associated sequence variants that were filtered out in this study did not have a significant impact on the downstream PNRSV diversity analysis.

The error detection and filtering process that was used in this study could be adapted to other similar studies. However it is possible that the filtered variants, including some with non-coding sequences could be real [82]. The possibility also exists that some sequence variants that do not contain non-coding sequence artefacts of PCR amplification and sequencing, which are more difficult to distinguish from true biological variants, were missed during filtering and remained in the dataset.

This study explored the usefulness of amplicon NGS as an alternative to traditional cloning and Sanger sequencing to determine the genetic diversity of PNRSV in 53 *Prunus* plant samples from Australia. Based on the findings presented, we were able to propose a definition of a PNRSV genetic strain and determine the number of PNRSV genetic strains present in plant samples with sequence variants belonging to a single phylo-group in each RNA component. However, the complexity associated with variants occurring in multiple phylo-groups for RNA1, RNA2 and/or RNA3 made it difficult to determine the number of strains of PNRSV in these plant samples. Never the less the analytical approach described here is multi-dimensional and applicable to both monopartite and multipartite genome viruses, and may assist biosecurity agencies in defining virus strains of quarantine significance.

## Supporting information

S1 Table The origin of each *Prunus* species sample used in this study. (XLSX)

S2 Table *Prunus necrotic ringspot virus* (PNRSV) type isolate sequences used for phylogenetic analysis for each RNA segment, the origin, host, phylo-groups and their corresponding GenBank accession numbers. (XLSX)

S3 Table The number of raw sequences reads generated from the next generation sequencing of *Prunus necrotic ringspot virus* (PNRSV) RNA1, RNA2 and RNA3 amplicons from each of the 53 plant samples, the number of reads remaining after trimming and the percentage read depth and the totals of each category. (XLSX)

S4 Table The total number of reads generated from transcribed RNA amplicon control triplicates and the resulting total number of clusters, singletons and stop codons from clustering at 100% identity. The percentage of error-associated reads of each triplicate and their average are also shown.
(XLSX)

S5 Table The total number of reads generated from transcribed RNA amplicon control triplicates and the resulting total number of clusters at 100% identity, the total number of nucleotide bases in all the clusters and the total number of error-associated nucleotide bases. The percentage amplicon error rate for each triplicate control and their average are also shown.
(XLSX)

S6 Table The number of amplicons clusters resulting from grouping of *Prunus necrotic ringspot virus* (PNRSV) RNA1 next generation sequencing amplicon reads of each of the 53 plant samples at 100% identity, and the number of singletons and non-coding clusters. The number of error-associated reads based on the 37% error rate and the final number of amplicon clusters and range after filtering.
(XLSX)

S7 Table The number of amplicons clusters resulting from grouping of *Prunus necrotic ringspot virus* (PNRSV) RNA2 next generation sequencing amplicon reads of each of the 53 plant samples at 100% identity, and the number of singletons and non-coding clusters. The number of error-associated reads based on the 37% error rate and the final number of amplicon clusters and range after filtering.
(XLSX)

S8 Table The number of amplicons clusters resulting from grouping of *Prunus necrotic ringspot virus* (PNRSV) RNA3 next generation sequencing amplicon reads of each of the 53 plant samples at 100% identity, and the number of singletons and non-coding clusters. The number of error-associated reads based on the 37% error rate and the final number of amplicon clusters and range after filtering.
(XLSX)

S9 Table The number of nucleotide variant positions and the resulting number synonymous (Syn.) and non-synonymous (Non-syn.) mutations observed from variant calling and annotation of *Prunus necrotic ringspot virus* (PNRSV) RNA1, 2 and 3 sequence variant clusters. (XLSX)

S10 Table The number of non-synonymous (Non-syn.) mutations and the predicted effect on the resulting amino acid sequence of *Prunus necrotic ringspot virus* (PNRSV) RNA1, 2 and 3 sequence variant clusters. (XLS)

## References

[1] García-Arenal F, Fraile A, Malpica JM. Variability and genetic structure of plant virus populations. Annu Rev Phytopathol. 2001;39(1):157–86. 11701863 10.1146/annurev.phyto.39.1.157

[2] Domingo E. Quasispecies and the implications for virus persistence and escape. Clinical and diagnostic virology. 1998;10(2–3):97–101. 9741634 10.1016/S0928-0197(98)00032-4 PMC7135314

[3] Eigen M, Schuster P. The Hypercycle. A Principle of Natural Self-Organisation. Naturwissenschaften. 1977;64(11):541–65. 593400 10.1007/BF00450633

[4] García-Arenal F, Fraile A, Malpica JM. Variation and evolution of plant virus populations. Int J Microbiol. 2003;6(4):225–32. doi: 10.1007/s10123-003-0142-z 13680390 10.1007/s10123-003-0142-z

[5] Lf Chen, Rojas M, Kon T, Gamby K, Xoconostle‐Cazares B, Gilbertson RL. A severe symptom phenotype in tomato in Mali is caused by a reassortant between a novel recombinant begomovirus (Tomato yellow leaf curl Mali virus) and a betasatellite. Mol Plant Pathol. 2009;10(3):415–30. doi: 10.1111/j.1364-3703.2009.00541.x 19400843 10.1111/j.1364-3703.2009.00541.x PMC6640326

[6] Pita J, Fondong V, Sangare A, Otim-Nape G, Ogwal S, Fauquet C. Recombination, pseudorecombination and synergism of geminiviruses are determinant keys to the epidemic of severe cassava mosaic disease in Uganda. J Gen Virol. 2001;82(3):655–65. 11172108 10.1099/0022-1317-82-3-655

[7] Ince WL, Gueye-Mbaye A, Bennink JR, Yewdell JW. Reassortment complements spontaneous mutation in influenza A virus NP and M1 genes to accelerate adaptation to a new host. J Virol. 2013;87(8):4330–8. doi: 10.1128/JVI.02749-12 23365443 10.1128/JVI.02749-12 PMC3624365

[8] Sanger F, Nicklen S, Coulson AR. DNA sequencing with chain-terminating inhibitors. Proc Natl Acad Sci. 1977;74(12):5463–7. 271968 10.1073/pnas.74.12.5463 PMC431765

[9] Cohen SN, Chang AC, Boyer HW, Helling RB. Construction of biologically functional bacterial plasmids in vitro. Proc Natl Acad Sci. 1973;70(11):3240–4. 4594039 10.1073/pnas.70.11.3240 PMC427208

[10] Cui H, Liu H, Chen J, Zhou J, Qu L, Su J, et al Genetic diversity of Prunus necrotic ringspot virus infecting stone fruit trees grown at seven regions in China and differentiation of three phylogroups by multiplex RT-PCR. Crop Protect. 2015;74:30–6.

[11] Beerenwinkel N, Zagordi O. Ultra-deep sequencing for the analysis of viral populations. Curr Opin Virol. 2011;1(5):413–8. doi: 10.1016/j.coviro.2011.07.008 22440844 10.1016/j.coviro.2011.07.008

[12] Barzon L, Lavezzo E, Costanzi G, Franchin E, Toppo S, Palù G. Next-generation sequencing technologies in diagnostic virology. J Clin Virol. 2013;58(2):346–50. doi: 10.1016/j.jcv.2013.03.003 23523339 10.1016/j.jcv.2013.03.003

[13] Adams IP, Glover RH, Monger WA, Mumford R, Jackeviciene E, Navalinskiene M, et al Next-generation sequencing and metagenomic analysis: a universal diagnostic tool in plant virology. Mol Plant Pathol. 2009;10(4):537–45. doi: 10.1111/j.1364-3703.2009.00545.x 19523106 10.1111/j.1364-3703.2009.00545.x PMC6640393

[14] Wu Q, Ding S-W, Zhang Y, Zhu S. Identification of Viruses and Viroids by Next-Generation Sequencing and Homology-Dependent and Homology-Independent Algorithms. Annu Rev Phytopathol. 2015;53:425–44. doi: 10.1146/annurev-phyto-080614-120030 26047558 10.1146/annurev-phyto-080614-120030

[15] Radford AD, Chapman D, Dixon L, Chantrey J, Darby AC, Hall N. Application of next-generation sequencing technologies in virology. J Gen Virol. 2012;93(Pt 9):1853–68. doi: 10.1099/vir.0.043182-0 22647373 10.1099/vir.0.043182-0 PMC3709572

[16] Prabha K, Baranwal V, Jain R. Applications of next generation high throughput sequencing technologies in characterization, discovery and molecular interaction of plant viruses. Indian J Virol. 2013;24(2):157–65. doi: 10.1007/s13337-013-0133-4 24426271 10.1007/s13337-013-0133-4 PMC3784914

[17] Marston DA, McElhinney LM, Ellis RJ, Horton DL, Wise EL, Leech SL, et al Next generation sequencing of viral RNA genomes. BMC genomics. 2013;14(1):1. 23822119 10.1186/1471-2164-14-444 PMC3708773

[18] Hall RJ, Wang J, Todd AK, Bissielo AB, Yen S, Strydom H, et al Evaluation of rapid and simple techniques for the enrichment of viruses prior to metagenomic virus discovery. J Virol Methods. 2014;195:194–204. doi: 10.1016/j.jviromet.2013.08.035 24036074 10.1016/j.jviromet.2013.08.035 PMC7113663

[19] Eriksson N, Pachter L, Mitsuya Y, Rhee S-Y, Wang C, Gharizadeh B, et al Viral population estimation using pyrosequencing. PLoS Comp Biol. 2008. 10.1371/journal.pcbi.1000074 PMC2323617 18437230

[20] Wang C, Mitsuya Y, Gharizadeh B, Ronaghi M, Shafer RW. Characterization of mutation spectra with ultra-deep pyrosequencing: application to HIV-1 drug resistance. Genome Res. 2007;17(8):1195–201. doi: 10.1101/gr.6468307 17600086 10.1101/gr.6468307 PMC1933516

[21] Wright CF, Morelli MJ, Thébaud G, Knowles NJ, Herzyk P, Paton DJ, et al Beyond the consensus: dissecting within-host viral population diversity of foot-and-mouth disease virus by using next-generation genome sequencing. J Virol. 2011;85(5):2266–75. doi: 10.1128/JVI.01396-10 21159860 10.1128/JVI.01396-10 PMC3067773

[22] McElroy K, Thomas T, Luciani F. Deep sequencing of evolving pathogen populations: applications, errors, and bioinformatic solutions. Microb Inform Exp. 2014;4(1):1 doi: 10.1186/2042-5783-4-1 24428920 10.1186/2042-5783-4-1 PMC3902414

[23] Tugume AK, Mukasa SB, Kalkkinen N, Valkonen JP. Recombination and selection pressure in the ipomovirus sweet potato mild mottle virus (Potyviridae) in wild species and cultivated sweetpotato in the centre of evolution in East Africa. J Gen Virol. 2010;91(4):1092–108. 19923261 10.1099/vir.0.016089-0

[24] Fabre F, Montarry J, Coville J, Senoussi R, Simon V, Moury B. Modelling the evolutionary dynamics of viruses within their hosts: a case study using high-throughput sequencing. PLoS Pathog. 2012;8(4):e1002654 doi: 10.1371/journal.ppat.1002654 22532800 10.1371/journal.ppat.1002654 PMC3330117

[25] Martínez F, Lafforgue G, Morelli MJ, González-Candelas F, Chua N-H, Daròs J-A, et al Ultradeep sequencing analysis of population dynamics of virus escape mutants in RNAi-mediated resistant plants. Mol Biol Evol. 2012;29(11):3297–307. doi: 10.1093/molbev/mss135 22593223 10.1093/molbev/mss135 PMC7187544

[26] Guo D, Maiss E, Adam G, Casper R. Prunus necrotic ringspot ilarvirus: nucleotide sequence of RNA3 and the relationship to other ilarviruses based on coat protein comparison. J Gen Virol. 1995;76(5):1073–9. 7730792 10.1099/0022-1317-76-5-1073

[27] Sánchez-Navarro J, Pallás V. Evolutionary relationships in the ilarviruses: nucleotide sequence of prunus necrotic ringspot virus RNA 3. Arch Virol. 1997;142(4):749–63. 9170502 10.1007/s007050050116

[28] Mink G, Howell W, Cole A, Regev S. Three serotypes of Prunus necrotic ringspot virus isolated from rugose mosaic-diseased sweet cherry trees in Washington. Plant Dis. 1987;71(1):91–3.

[29] Hammond RW, Crosslin JM. Virulence and molecular polymorphism of Prunus necrotic ringspot virus isolates. J Gen Virol. 1998;79(7):1815–23. 9680147 10.1099/0022-1317-79-7-1815

[30] Aparicio F, Myrta A, Di Terlizzi B, Pallás V. Molecular variability among isolates of Prunus necrotic ringspot virus from different Prunus spp. Phytopathology. 1999;89(11):991–9. doi: 10.1094/PHYTO.1999.89.11.991 18944653 10.1094/PHYTO.1999.89.11.991

[31] Fiore N, Fajardo TV, Prodan S, Herranz MC, Aparicio F, Montealegre J, et al Genetic diversity of the movement and coat protein genes of South American isolates of Prunus necrotic ringspot virus. Arch Virol. 2008;153(5):909–19. doi: 10.1007/s00705-008-0066-1 18365129 10.1007/s00705-008-0066-1

[32] Fajardo TVM, Nascimento MB, Eiras M, Nickel O, Pio-Ribeiro G. Molecular characterization of Prunus necrotic ringspot virus isolated from rose in Brazil. Ciência Rural. 2015;45(12):2197–200.

[33] MacKenzie DJ, McLean MA, Mukerji S, Green M. Improved RNA Extraction from Woody Plants for the Detection of Viral Pathogens by Reverse Transcription-Polymerase Chain Reaction. Plant Dis Rep. 1997; 81 (2):222–6. 10.1094/PDIS.1997.81.2.222 30870901

[34] Illumina. Preparing Libraries for Sequencing on the MiSeq® 2013. Available from: https://support.illumina.com/content/dam/illumina-support/documents/documentation/system_documentation/miseq/preparing-libraries-for-sequencing-on-miseq-15039740-d.pdf.

[35] Krueger F. Trim Galore! 2012. Available from: www.bioinformatics.babraham.ac.uk/projects/trim_galore.

[36] Zhang J, Kobert K, Flouri T, Stamatakis A. PEAR: a fast and accurate Illumina Paired-End reAd mergeR. Bioinformatics. 2014;30(5):614–20. doi: 10.1093/bioinformatics/btt593 24142950 10.1093/bioinformatics/btt593 PMC3933873

[37] Pearson WR, Wood T, Zhang Z, Miller W. Comparison of DNA sequences with protein sequences. Genomics. 1997;46(1):24–36. doi: 10.1006/geno.1997.4995 9403055 10.1006/geno.1997.4995

[38] Altschul SF, Madden TL, Schäffer AA, Zhang J, Zhang Z, Miller W, et al Gapped BLAST and PSI-BLAST: a new generation of protein database search programs. Nucleic Acids Res. 1997;25(17):3389–402. 9254694 10.1093/nar/25.17.3389 PMC146917

[39] Edgar RC. MUSCLE: multiple sequence alignment with high accuracy and high throughput. Nucleic Acids Res. 2004;32(5):1792–7. doi: 10.1093/nar/gkh340 15034147 10.1093/nar/gkh340 PMC390337

[40] Martin M. Cutadapt removes adapter sequences from high-throughput sequencing reads. EMBnet journal. 2011;17(1):pp. 10–2.

[41] Edgar RC. Search and clustering orders of magnitude faster than BLAST. Bioinformatics. 2010;26(19):2460–1. doi: 10.1093/bioinformatics/btq461 20709691 10.1093/bioinformatics/btq461

[42] Rice P, Longden I, Bleasby A. EMBOSS: the European molecular biology open software suite. Trends Genet. 2000;16(6):276–7. 10827456 10.1016/s0168-9525(00)02024-2

[43] Stamatakis A. RAxML version 8: a tool for phylogenetic analysis and post-analysis of large phylogenies. Bioinformatics. 2014;30(9):1312–3. doi: 10.1093/bioinformatics/btu033 24451623 10.1093/bioinformatics/btu033 PMC3998144

[44] Andrew R. FigTree 2009. Available from: http://tree.bio.ed.ac.uk/software/.

[45] Muhire BM, Varsani A, Martin DP. SDT: a virus classification tool based on pairwise sequence alignment and identity calculation. PLoS ONE. 2014. 10.1371/journal.pone.0108277 PMC4178126 25259891

[46] Bandelt H-J, Forster P, Röhl A. Median-joining networks for inferring intraspecific phylogenies. Mol Biol Evol. 1999;16(1):37–48. 10331250 10.1093/oxfordjournals.molbev.a026036

[47] Li H, Durbin R. Fast and accurate short read alignment with Burrows–Wheeler transform. Bioinformatics. 2009;25(14):1754–60. doi: 10.1093/bioinformatics/btp324 19451168 10.1093/bioinformatics/btp324 PMC2705234

[48] Li H, Handsaker B, Wysoker A, Fennell T, Ruan J, Homer N, et al The sequence alignment/map format and SAMtools. Bioinformatics. 2009;25(16):2078–9. doi: 10.1093/bioinformatics/btp352 19505943 10.1093/bioinformatics/btp352 PMC2723002

[49] Garrison E, Marth G. Haplotype-based variant detection from short-read sequencing. arXiv preprint arXiv:12073907. 2012.

[50] Cingolani P, Platts A, Wang LL, Coon M, Nguyen T, Wang L, et al A program for annotating and predicting the effects of single nucleotide polymorphisms, SnpEff: SNPs in the genome of Drosophila melanogaster strain. Fly. 2012;6(2):80–92. doi: 10.4161/fly.19695 22728672 10.4161/fly.19695 PMC3679285

[51] Aparicio F, Pallás V. The molecular variability analysis of the RNA 3 of fifteen isolates of Prunus necrotic ringspot virus sheds light on the minimal requirements for the synthesis of its subgenomic RNA. Virus Genes. 2002;25(1):75–84. 12206311 10.1023/a:1020126309692

[52] Bergua M, Luis-Arteaga M, Escriu F. Genetic diversity, reassortment, and recombination in Alfalfa mosaic virus population in Spain. Phytopathology. 2014;104(11):1241–50. doi: 10.1094/PHYTO-11-13-0309-R 24779352 10.1094/PHYTO-11-13-0309-R

[53] Roossinck MJ. Evolutionary history of Cucumber mosaic virus deduced by phylogenetic analyses. J Virol. 2002;76(7):3382–7. doi: 10.1128/JVI.76.7.3382-3387.2002 11884564 10.1128/JVI.76.7.3382-3387.2002 PMC136033

[54] Van Regenmortel MH. Viruses are real, virus species are man-made, taxonomic constructions. Arch Virol. 2003;148(12):2481–8. doi: 10.1007/s00705-003-0246-y 14648301 10.1007/s00705-003-0246-y

[55] Pringle C. The 20th meeting of the executive committee of the international committee on virus taxonomy. Arch Virol. 1991;119(3):303–4.

[56] Hull R. Plant virology: Academic press; 2013.

[57] Randles J, Ogle H. Viruses and viroids as agents of plant disease Plant Pathogens and Plant Diseases Brown JF, Ogle HJ editor Australia: Rockvale Publication 1997:104–26.

[58] Fauquet C, Stanley J. Revising the way we conceive and name viruses below the species level: a review of geminivirus taxonomy calls for new standardized isolate descriptors. Arch Virol. 2005;150(10):2151–79. doi: 10.1007/s00705-005-0583-0 16132185 10.1007/s00705-005-0583-0

[59] Kuhn JH, Bao Y, Bavari S, Becker S, Bradfute S, Brister JR, et al Virus nomenclature below the species level: a standardized nomenclature for laboratory animal-adapted strains and variants of viruses assigned to the family Filoviridae. Arch Virol. 2013;158(6):1425–32. doi: 10.1007/s00705-012-1594-2 23358612 10.1007/s00705-012-1594-2 PMC3669655

[60] Van Regenmortel M. Virus species and virus identification: past and current controversies. Infect Genet Evol. 2007;7(1):133–44. doi: 10.1016/j.meegid.2006.04.002 16713373 10.1016/j.meegid.2006.04.002

[61] Simmonds P. Methods for virus classification and the challenge of incorporating metagenomic sequence data. J Gen Virol. 2015;96(6):1193–206. 26068186 10.1099/jgv.0.000016

[62] Iranzo J, Manrubia SC. Evolutionary dynamics of genome segmentation in multipartite viruses. Proc R Soc Lond [Biol]. 2012;279(1743):3812–9. 10.1098/rspb.2012.1086 PMC3415918 22764164

[63] van Vloten-Doting L, Bol J-F, Cornelissen B. Plant-virus-based vectors for gene transfer will be of limited use because of the high error frequency during viral RNA synthesis. Plant Mol Biol. 1985;4(5):323–6. doi: 10.1007/BF02418253 24310884 10.1007/BF02418253

[64] Scott S, Zimmerman M, Ge X, MacKenzie D. The coat proteins and putative movement proteins of isolates of Prunus necrotic ringspot virus from different host species and geographic origins are extensively conserved. Eur J Plant Pathol. 1998;104(2):155–61.

[65] Sánchez-Navarro JA, Zwart MP, Elena SF. Effects of the number of genome segments on primary and systemic infections with a multipartite plant RNA virus. J Virol. 2013;87(19):10805–15. doi: 10.1128/JVI.01402-13 23903837 10.1128/JVI.01402-13 PMC3807391

[66] Koonin EV. The phylogeny of RNA-dependent RNA polymerases of positive-strand RNA viruses. J Gen Virol. 1991;72(9):2197–206. 1895057 10.1099/0022-1317-72-9-2197

[67] Pallas V, Aparicio F, Herranz M, Amari K, Sanchez-Pina M, Myrta A, et al Ilarviruses of Prunus spp.: A continued concern for fruit trees. Phytopathology. 2012;102(12):1108–20. doi: 10.1094/PHYTO-02-12-0023-RVW 23148725 10.1094/PHYTO-02-12-0023-RVW

[68] Di Terlizzi B, Skrzeczkowski L, Mink G, Scott S, Zimmerman M. The RNA 5 of Prunus necrotic ringspot virus is a biologically inactive copy of the 3′-UTR of the genomic RNA 3. Arch Virol. 2001;146(4):825–33. 11402868 10.1007/s007050170151

[69] Vlot AC, Menard A, Bol JF. Role of the alfalfa mosaic virus methyltransferase-like domain in negative-strand RNA synthesis. J Virol. 2002;76(22):11321–8. doi: 10.1128/JVI.76.22.11321-11328.2002 12388692 10.1128/JVI.76.22.11321-11328.2002 PMC136773

[70] Jridi C, Martin J-F, Marie-Jeanne V, Labonne G, Blanc S. Distinct viral populations differentiate and evolve independently in a single perennial host plant. J Virol. 2006;80(5):2349–57. doi: 10.1128/JVI.80.5.2349-2357.2006 16474141 10.1128/JVI.80.5.2349-2357.2006 PMC1395380

[71] Dunham J, Simmons H, Holmes E, Stephenson A. Analysis of viral (zucchini yellow mosaic virus) genetic diversity during systemic movement through a Cucurbita pepo vine. Virus Res. 2014;191:172–9. doi: 10.1016/j.virusres.2014.07.030 25107623 10.1016/j.virusres.2014.07.030 PMC4176823

[72] Dickie IA. Insidious effects of sequencing errors on perceived diversity in molecular surveys. New Phytologist. 2010;188(4):916–8. doi: 10.1111/j.1469-8137.2010.03473.x 20854395 10.1111/j.1469-8137.2010.03473.x

[73] Zhou J, Wu L, Deng Y, Zhi X, Jiang Y-H, Tu Q, et al Reproducibility and quantitation of amplicon sequencing-based detection. The ISME journal. 2011;5(8):1303–13. doi: 10.1038/ismej.2011.11 21346791 10.1038/ismej.2011.11 PMC3146266

[74] Brodin J, Mild M, Hedskog C, Sherwood E, Leitner T, Andersson B, et al PCR-induced transitions are the major source of error in cleaned ultra-deep pyrosequencing data. PloS one. 2013;8(7):e70388 doi: 10.1371/journal.pone.0070388 23894647 10.1371/journal.pone.0070388 PMC3720931

[75] Kunin V, Engelbrektson A, Ochman H, Hugenholtz P. Wrinkles in the rare biosphere: pyrosequencing errors can lead to artificial inflation of diversity estimates. Environ Microbiol. 2010;12(1):118–23. doi: 10.1111/j.1462-2920.2009.02051.x 19725865 10.1111/j.1462-2920.2009.02051.x

[76] Quince C, Lanzen A, Davenport RJ, Turnbaugh PJ. Removing noise from pyrosequenced amplicons. BMC bioinformatics. 2011;12(1):1. 21276213 10.1186/1471-2105-12-38 PMC3045300

[77] Zagordi O, Klein R, Däumer M, Beerenwinkel N. Error correction of next-generation sequencing data and reliable estimation of HIV quasispecies. Nucleic Acids Res. 2010;38(21):7400–9. doi: 10.1093/nar/gkq655 20671025 10.1093/nar/gkq655 PMC2995073

[78] Prabhakaran S, Rey M, Zagordi O, Beerenwinkel N, Roth V, editors. HIV-haplotype inference using a constraint-based dirichlet process mixture model. Machine Learning in Computational Biology (MLCB) NIPS Workshop; 2010. 10.1109/TCBB.2013.145 26355517

[79] Westbrooks K, Astrovskaya I, Campo D, Khudyakov Y, Berman P, Zelikovsky A. HCV quasispecies assembly using network flows Bioinformatics Research and Applications: Springer; 2008 p. 159–70.

[80] Prosperi MC, Salemi M. QuRe: software for viral quasispecies reconstruction from next-generation sequencing data. Bioinformatics. 2012;28(1):132–3. doi: 10.1093/bioinformatics/btr627 22088846 10.1093/bioinformatics/btr627 PMC3244773

[81] Schirmer M, Ijaz UZ, D'Amore R, Hall N, Sloan WT, Quince C. Insight into biases and sequencing errors for amplicon sequencing with the Illumina MiSeq platform. Nucleic acids research. 2015:gku1341. 10.1093/nar/gku1341 PMC4381044 25586220

[82] Orton RJ, Wright CF, Morelli MJ, King DJ, Paton DJ, King DP, et al Distinguishing low frequency mutations from RT-PCR and sequence errors in viral deep sequencing data. BMC genomics. 2015;16(1):1. 25886445 10.1186/s12864-015-1456-x PMC4425905

